# Letter to the editor: Headline stress disorder caused by Netnews during the outbreak of COVID‐19

**DOI:** 10.1111/hex.13055

**Published:** 2020-03-30

**Authors:** Mengyuan Dong, Jin Zheng

**Affiliations:** ^1^ Department of Urology The First Affiliated Hospital of China Medical University Shenyang China

To the editor:

In December 2019, an ongoing outbreak of a novel coronavirus (COVID‐19 or 2019‐nCoV) pneumonia hit Wuhan, a major city of China, and then quickly spread to other provinces/regions of China and even the whole world. It has attracted worldwide attention, and for a long time, the news coverage was overwhelming. The news coverage made the general public feel dazed and even plunged into anxiety and panic. The use of instant‐messaging technology and mobile phones makes the news spread faster and exacerbates the anxiety and panic of public. The psychological disorder caused by too many news coverage was named as ‘headline stress disorder’. It^1^ was first defined by psychologist Dr Steven Stosny as a high emotional response to endless reports from the news media, such as feeling anxiety and stress. Although it is not a medical diagnosis, the continued anxiety or stress may cause physical functionals disorders, including palpitation, chest tightness and insomnia, and further progression may lead to physical and mental diseases, such as anxiety disorders, depression disorders, endocrine disorders and hypertension.^2^, ^3^ On 30 September 2014, after the first laboratory‐diagnosed Ebola virus was diagnosed in the United States, a media firestorm erupted with constant updates of the Ebola ‘crisis’.^4^ The public anxiety and panic ensued after the report was frequently attributed to the media's dramatized and sensational coverage.^5^ Subsequently, media reports from unauthorized sources during the ongoing monkeypox outbreak in Nigeria were sensationalized and led to increased anxiety in the population.^6^ The result of this type of coverage could lead to stress and panic among the general public. This is an important issue because concerns about media coverage during a health crisis may likely re‐emerge.^7^

During the outbreak of COVID‐19, we conducted an online investigation on the perception of online news in China. A total of 1206 general public participated in the survey, including 478 males and 728 females. The results are listed in Table 1. From the results, we known that most of general public thought unofficial news about COVID‐19 was too much and less reliable, and most of them suggested government to take some control of unofficial news coverage and release COVID‐19 related information timely.

**Table 1:** Publics’ attitude to online news coverage during the outbreak of COVID‐19

Questions	Strongly disagree	Disagree	Uncertainty	Agree	Strongly agree
1. I think people now are in a hurry to see a doctor	143 (11.9%)	270 (22.4%)	282 (23.4%)	371 (30.8%)	140 (11.6%)
2. I think the government should publish information about COVID‐19 every day	22 (1.8%)	41 (3.4%)	60 (5.0%)	220 (18.2%)	863 (71.6%)
3. I think the government should take measures to restrict population movements	31 (2.6%)	44 (3.6%)	64 (5.3%)	286 (23.7%)	781 (64.8%)
4. I think there should be some control over the information about COVID‐19	181 (15.0%)	232 (19.2%)	141 (11.7%）	266 (22.1%)	386 (32.0%)
5. I think there are too many reports on COVID‐19	274 (22.7%)	344 (28.5%)	182 (15.1%)	250 (20.7%)	156 (12.9%)
6. I think the unofficial information about COVID‐19 is not credible	96 (8.0%)	130 (25.5%)	307 (25.5%)	348 (28.9%)	325 (26.9%)
7. I think the government should strengthen the control of unofficial information about COVID‐19	51 (4.2%)	102 (8.5%)	161 (13.3%)	444 (36.8%)	448 (37.1%)

Media organizations play an important role in the dissemination of news pertaining to public health crises, and media coverage has a direct or indirect impact on public behaviours. On the one hand, media publicity could increase public's knowledge and prevention tips about COVID‐19. For example, our survey showed that 88.5% of residents thought that people should be restricted and stayed at home as possible during the current outbreak of COVID‐19. On the other hand, exaggerated news reports would bring panic, stress and anxiety to public. In this survey, 42.4% participants thought public were too nervous and seeking for any possible way for prevent or treatment. For example, a report entitled ' SHUANGHUANGLIAN which is traditional Chinese drug could inhibits novel coronavirus virus' appeared on January 31 on major websites and social platforms, immediately, public flocks to pharmacies and online stores to snap up related medicines. The next day, however, it proved to be an inflated report of the efficacy of the drug. Therefore, all media reporting on public health issues, especially in major health crises, should base on reality without exaggeration and falsehood.

The general public is easily affected by the news coverage published by the media. How to shield from headline stress disorder is an important issue for public. First, we suggested public to be prudent and critical about the unofficial report. Second, we recommend that the public arrange reasonable time for reading news information. Third, we recommend that the public focus on what they could change now instead of pay much attention on news. Indeed, public health institutes could combat misinformation by frequently updating key facts, available on their official media platforms for clarification. Finally, we suggested that the government or the news media industry organizations ban relating laws or regulations to regulate the news coverage, so as not to cause public panic and headline stress disorder in the event of a public emergency.

## CONFLICT OF INTEREST

The authors declare that they have no competing interests.

## Data Availability

The data that support the findings of this study are available from the corresponding author upon reasonable request.
